# The Detection of Yarn Roll’s Margin in Complex Background

**DOI:** 10.3390/s23041993

**Published:** 2023-02-10

**Authors:** Junru Wang, Zhiwei Shi, Weimin Shi, Hongpeng Wang

**Affiliations:** School of Mechanical Engineering, Zhejiang Sci-Tech University, Hangzhou 310018, China

**Keywords:** detection of yarn roll’s margin, computer vision, Yolo model, contours detection, Kalman filter

## Abstract

Online detection of yarn roll’s margin is one of the key issues in textile automation, which is related to the speed and scheduling of bobbin (empty yarn roll) replacement. The actual industrial site is characterized by uneven lighting, restricted shooting angles, diverse yarn colors and cylinder yarn types, and complex backgrounds. Due to the above characteristics, the neural network detection error is large, and the contour detection extraction edge accuracy is low. In this paper, an improved neural network algorithm is proposed, and the improved Yolo algorithm and the contour detection algorithm are integrated. First, the image is entered in the Yolo model to detect each yarn roll and its dimensions; second, the contour and dimensions of each yarn roll are accurately detected based on Yolo; third, the diameter of the yarn rolls detected by Yolo and the contour detection algorithm are fused, and then the length of the yarn rolls and the edges of the yarn rolls are calculated as measurements; finally, in order to completely eliminate the error detection, the yarn consumption speed is used to estimate the residual yarn volume and the measured and estimated values are fused using a Kalman filter. This method overcomes the effects of complex backgrounds and illumination while being applicable to different types of yarn rolls. It is experimentally verified that the average measurement error of the cylinder yarn diameter is less than 8.6 mm, and the measurement error of the cylinder yarn length does not exceed 3 cm.

## 1. Introduction

Automation and intelligence are commonly utilized in the textile industry, throughout the entire textile process [1]. Due to environmental constraints at the industrial sites, the camera can only shoot from the cross-section of the cylinder yarn, and the calculation of the cylinder yarn length is, therefore, difficult. The inconsistent lighting conditions in the plant, as well as the complex internal background, can interfere with the detection. Moreover, the inconsistency of the cylinder yarn color, type, etc., requires that the detection algorithm be more versatile. In industrial applications, in order to replace the scheduling arrangements for the replacement of the cylinder yarn, detection of empty cylinders, identification of bobbins (empty yarn rolls), and detection of yarn roll residuals are key steps [2,3]. To solve this problem, this paper proposes a computer vision-based method for online detection of yarn roll residuals [4,5,6,7,8,9]. The method combines conventional computer vision and deep learning to detect the major dimensions of yarn rolls. According to experimental tests, the average measurement error for a conical yarn roll diameter is 6 mm, while it is no more than 12 mm for a cylindrical yarn roll diameter; yarn roll length is no more than 3 cm when the margin is large, the latter meeting the requirements of enterprises and helping automate the whole textile process.

**Figure 1:**
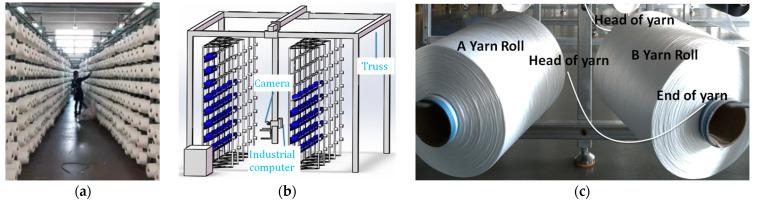
Environment. (**a**) Production environment. (**b**) Truss and vision system. (**c**) Yarn’s head and end is connected.

In many small and medium-sized enterprises, workers manually place every yarn roll on supports, as shown in Figure 1a. The camera, industrial computer, and bobbin replacement device are installed on the truss. The truss drives the vision system to detect every yarn roll’s margin and judge whether the yarn roll is exhausted. If it is exhausted, the empty bobbin will be replaced by the bobbin replacement device. The structure of the truss system is shown in Figure 1b. In order to ensure that the spinning is not interrupted during the bobbin changing process, the head and end of two adjacent yarn roll are connected (i.e., the thread head of yarn roll A is connected to the thread end of yarn roll B), as shown in Figure 1c. In such a scenario, several problems need to be solved for the automatic detection process to be successful: (1) the detection of empty yarn rolls must be accurate; (2) in a textile factory, there are many empty bobbins at the same time and the empty bobbin B with a margin smaller than that of bobbin A must be replaced first. In order to improve the accuracy of empty bobbin detection and solve the scheduling problem, it is necessary to detect the margin of yarn rolls.

**Figure 2:**
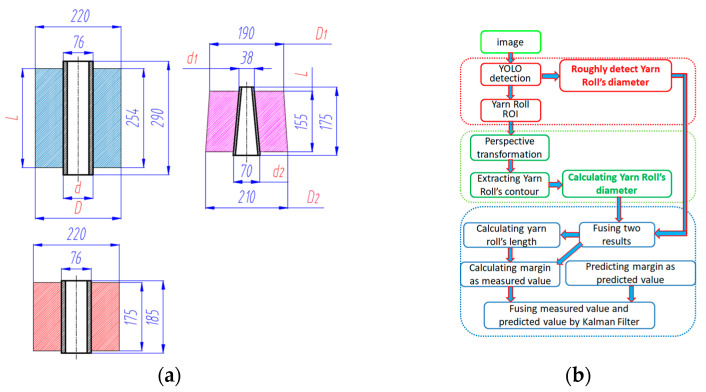
Yarn roll’s type and algorithm flow. (**a**) Yarn roll’s type and size. (**b**) Algorithm flow.

According to our research, there are three types of yarn roll commonly used by enterprises, as shown in Figure 2. The margin of a yarn roll is defined as shown in Formulas (1) and (2). Formula (1) is used to calculate the margin of a conic yarn roll. $D_{1}$ is yarn roll’s top surface diameter,$\;D_{2}$ is the yarn roll’s bottom surface diameter, $d_{1}$ is the bobbin’s top surface diameter,$\;d_{1}$ is the bobbin’s bottom surface diameter, l is the yarn roll’s length. Formula (2) is used to calculate the margin of a cylindrical yarn roll. D is the yarn roll’s diameter, d is the bobbin’s diameter, l is the yarn roll’s length, as shown in Figure 2a. Therefore, it is necessary to detect three quantities: bobbin diameter d, yarn roll diameter D, and yarn roll length l.

(1) $$V=\frac{\pi }{3}\left(\frac{D_{2}{}^{2}}{4}+\frac{D_{1}{}^{2}}{4}+\frac{D_{1}D_{2}}{4}\right)l-\frac{\pi }{3}\left(\frac{d_{2}{}^{2}}{4}+\frac{d_{1}{}^{2}}{4}+\frac{d_{1}d_{2}}{4}\right)l$$

(2) $$V=\frac{\pi }{4}\left(D^{2}-d^{2}\right)l$$

According to the yarn roll’s layout and reliability requirements, there are several challenges to the margin detection process: (1) the illumination is unstable and the background is complex, as the image background contains other yarn rolls, which will interfere with detection of the target yarn roll; (2) the algorithm needs to be applicable to various types of yarn roll. 

In order to solve the above problems and challenges, this paper proposes a method that can detect the margin of a yarn roll accurately. Firstly, every yarn roll and its pixel diameter are roughly detected by the Yolo model. Then, every ROI image of each yarn roll is further processed to extract the yarn roll’s inner and outer contour. Thirdly, the two detected diameters (detected by Yolo and contours) are fused together and the length of every detected yarn roll is calculated. Fourthly, the margin of the yarn roll is calculated as a measured value, and the current margin of the yarn roll is predicted as a predicted value. Lastly, the measured value and the predicted value are fused by Kalman Filter. The whole algorithm flow is shown in Figure 2b. After verification, this method can effectively solve the existing problems.

## 2. Related Work

According to the literature, there are two main methods for measuring yarn roll dimensions: contact measurement technology [2,3,4,5] and non-contact measurement technology [10,11,12,13,14,15,16,17,18,19]. Contact measurement technology mainly uses various instruments or sensors to detect yarn roll’s size or features. Non-contact measurement technology mainly uses computer vision to extract color, edge, and other characteristics of yarn roll.

Contact measurement technology measures some characteristics of yarn, such as tension, through sensors during the manufacturing process, or entails putting the yarn roll into some equipment to measure the external dimensions of the yarn roll. Imae M, Iwade T, and Shintani Y proposed a yarn detection method [4] based on strain sensor. However, the utilized instruments may cause damage to the yarn roll.

Non-contact measurement technology mainly uses computer vision to detect the yarn roll. It can be divided into monocular vision method, stereo vision method, and deep learning method [20,21,22,23,24,25,26,27,28].

(1) Monocular vision method: Du, C.J., and Sun, D.W. propose an automatic method [18] to estimate the surface area and volume of ham. They extract the ham through image-by-image segmentation and edge detection. This method cannot process complex background. Similarly, Jing, H., Li, D., and Duan, Q. present a method [19] to classify species of fish based on color and texture features using a multi-class support vector machine (MSVM). In the present paper, the yarn roll has different color and texture features, so this method cannot be used directly. This method requires pure background and is not very accurate for volume or class detection.
(2) Stereo vision method: compared with monocular camera, the stereo camera can measure an object’s three-dimensional information, and the measurement result is more accurate; it is also widely used in the industry. However, stereo camera needs to be calibrated before use, and the three-dimensional information is obtained through intrinsic and extrinsic matrices, a process that is more complicated. Molinier, T., Fofi, D., and Bingham, P.R. use two-vision systems to extract complementary 3D fire points [20]. The obtained data are projected in a same reference frame and used to build a global form of the fire front. From the obtained 3D fire points, a three-dimensional surface rendering is performed and the fire volume estimated. Sheng, J., Zhao, H., and Bu, P. propose a four-direction global matching with a cost volume update scheme [21] to cope with textureless regions and occlusion. Experimental results show that their method is highly efficient.
(3) There are some defects in the algorithms commonly used in edge detection, for example, the canny algorithm [29,30,31,32,33] uses Gaussian filtering for smoothing and noise reduction, which only considers the similarity of images in the spatial domain, and the filtering process leads to the loss of some useful weak edges. At the same time, the selection of the size of the convolution kernel of Gaussian filtering is influenced by human factors and, if the value is too small, the noise of the image cannot be effectively suppressed and the smoothed image is blurred. The canny algorithm uses a 2 × 2 size convolution kernel with two directions of horizontal and vertical detection, which is too small to extract the complete edge information, too sensitive to noise, and easily captures a pseudo-edge. The traditional canny algorithm relies on human experience to select the high and low thresholds, which cannot take into account the local feature information, and the uncertainty of the threshold value appears to be a certain error and lacks self-adaptability.
(4) Deep learning method: as an important branch of machine vision, deep learning technology has been undergoing rapid development in recent years and has been used more and more widely in the industrial field. Yolo [29,30,31,32] is a type of neural network which is used to detect the target online. It transforms the object detection into a regression problem. We can directly obtain the position in the image, the corresponding category of the object, and its confidence only through one inference. Yolo does not explicitly solve region proposal, but it integrates the process into the network, which reduces the operational complexity of the detection process and improves the efficiency of the algorithm to a certain extent.

## 3. Yarn Roll’s Margin Detection Method

As mentioned above, this paper mainly uses computer vision technology to detect yarn roll’s margin. Based on the characteristics of the edge detection algorithm, the following algorithm is used to detect the edges of the yarn roll in combination with the characteristics of the edges. The main algorithm is as follows.

### 3.1. Yolo Model

**Figure 3:**
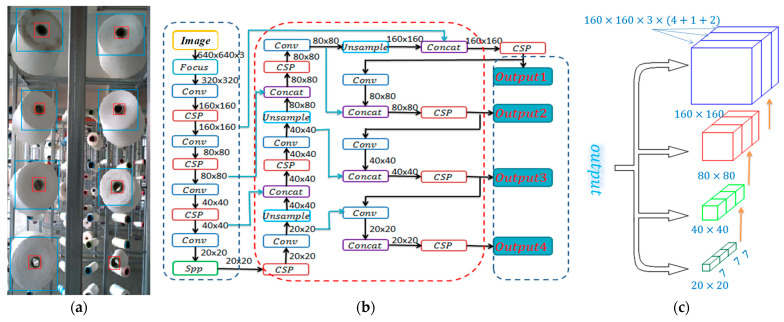
Yolo model. (**a**) Training sample. (**b**) The structure of network. (**c**) Model’s outputs.

There are four types of networks with different depths in yolov5 [34,35,36,37]. Network structure and depth can be modified through a configuration file. A configuration file is divided into three parts. The first part is the anchor box value. The second part is the backbone network, which is mainly used to extract features. The third part is the head layer, which mainly combines image features and transfers features to the prediction layer (output layer). The prediction layer is responsible for generating the output for the category of the target and its bounding box. We use a VOC dataset as a training sample. Target objects are the bobbin’s end surface (red box in Figure 3a) and the yarn roll’s end surface (blue box in Figure 3a).

In order to obtain a better detection effect, a detection layer is added and three anchor box values will be added correspondingly. The final network structure is shown in Figure 3b, and its output also has four groups, as shown in Figure 3c. There are only two types of targets in training samples. The model ultimately generates four matrices, their sizes being (i) 20 × 20 × 3 × 7, (ii) 40 × 40 × 3 × 7, (iii) 80 × 80 × 3 × 7, and (iv) 160 × 160 × 3 × 7, respectively.

**Figure 4:**
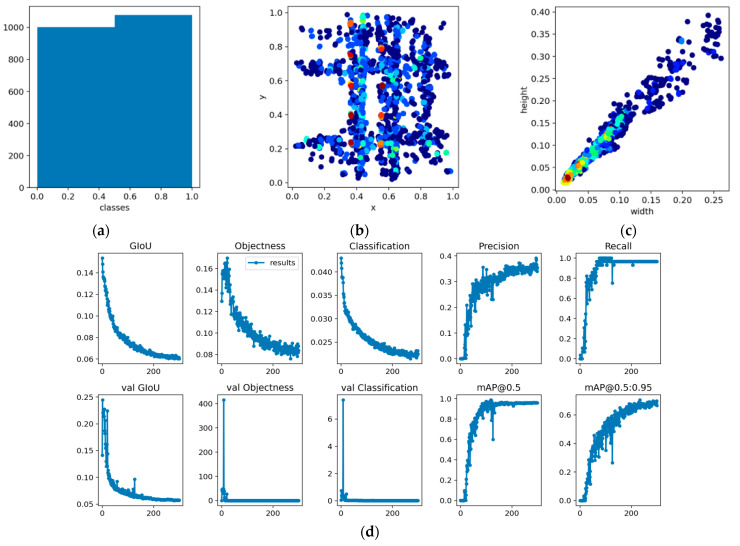
Sample analysis and model training results. (**a**) Samples number. (**b**) Objects coordinate distribution. (**c**) Statistical chart of sample box length-width ratio. (**d**) Training results.

The training results are shown in Figure 4. Figure 4a is a statistics chart of the sample number; Figure 4b refers to the coordinate distribution of targets in the image, with coordinates being normalized; Figure 4c is the length–width ratio of anchor boxes. It can be observed from this figure that the length–width ratio is close to 1:1. Figure 4d shows the training results, where GIOU represents the average value of the loss function: the smaller this value is, the more accurate the target box. Objectness is the average loss: the smaller the value, the more accurate the target detection. Classification refers to the classification accuracy: the smaller the classification, the more accurate the classification. Precision refers to the proportion of targets correctly detected by the model relative to the number of all targets detected by model: if the detection accuracy is 100%, it means that all targets detected by the model are correct, but this does not mean this model is good, because there may be undetected objects. Recall rate refers to the proportion of targets correctly detected by model relative to all targets. This evaluates the model’s ability to find all objects.

Figure 4a shows the sample size statistics, there are two types of target detection objects, namely, the end face of the cylinder yarn and the end face of the yarn cylinder, the number of which is about 1000 each and can reach nearly 10,000 by data enhancement. Figure 4b shows the coordinate distribution of the target objects (yarn end face and yarn end face center) in the image, the coordinates are normalized coordinates. 

**Figure 5:**
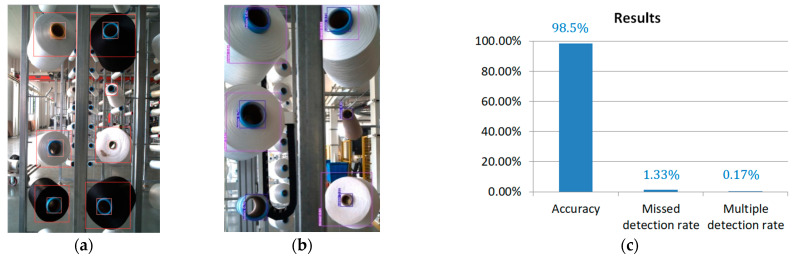
Yolo model detection results. (**a**) Detection results. (**b**) Detection results. (**c**) Measured error.

Results are shown in Figure 5. It can be seen from Figure 5a,b that the model only detects yarn rolls in the image foreground, eliminating the influence of yarn rolls in the background. We randomly collected 100 images, amounting to about 600 yarn rolls, and entered them into model. When the detection threshold is set to 0.5, the final detection result is shown in Figure 5c, where the detection accuracy rate is 98.5%, the missed detection rate is 1.33%, and the multiple detection rate of an object is 0.17%.

### 3.2. Contours Extraction and Process

**Figure 6:**
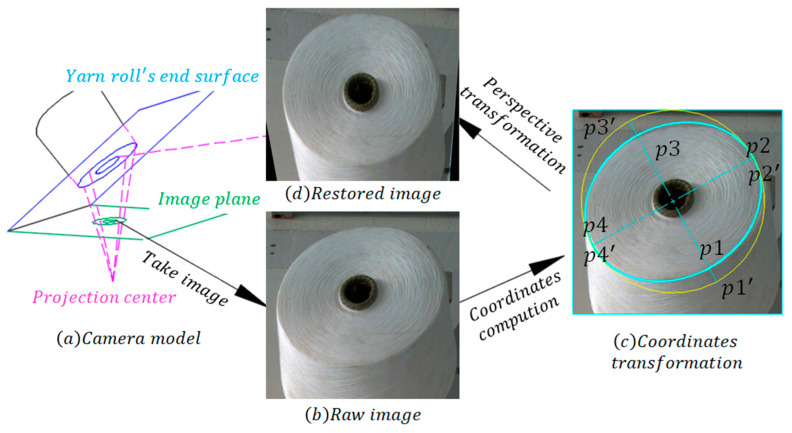
Perspective transformation process.

Through the above steps, the diameter of the yarn roll and of the bobbin are detected, roughly. We selected every yarn roll’s ROI images. Because the background is complex and the camera plane is not parallel to the bobbin’s end face plane, as shown in Figure 6a, it is difficult to extract the yarn roll’s contours accurately. Therefore, we processed ROI images through the following steps: (1) restoring ROI image by perspective transformation; (2) finding the yarn roll’s end surface center accurately through circle-filters; and, (3) processing and extracting the yarn roll’s contours.

#### 3.2.1. Perspective Transformation

In order to detect the yarn roll’s diameter accurately, the ROI image needs to be restored using perspective transformation [12]. There are 8 yarn rolls in one image. Therefore, we should calculate eight perspective transformation matrices in advance. The process is shown in Figure 6. For every yarn roll station, we need four original coordinates and its transformed coordinates to obtain a transformative matrix: the four original coordinates are the vertex coordinates $\left(P1,P2,P3,P4\right)$, while its transformed coordinates are $\left(P1^{\prime },P2^{\prime },P3^{\prime },P4^{\prime }\right)$, as shown in Figure 6c. Then, Formula (3) should be used to calculate the transformative matrix. $\left(u,v\right)$ are the original coordinates, w is 1, and $\left(x,y\right)$ are the transformed coordinates. Lastly, we restore the image by using the transformative matrix, as shown in Figure 6d.

(3) $$\left[\begin{array}{ccc} x^{\prime } & y^{\prime } & w^{\prime } \end{array}\right]=\left[\begin{array}{ccc} u & v & w \end{array}\right]\left[\begin{array}{ccc} a_{11} & a_{12} & a_{13}\\ a_{21} & a_{22} & a_{23}\\ a_{31} & a_{32} & a_{33} \end{array}\right]$$

#### 3.2.2. Detecting the Yarn Roll’s Center

**Figure 7:**
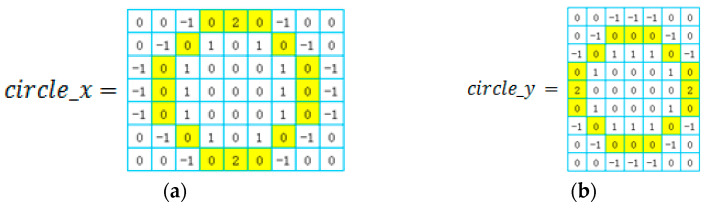
Circle filters.

The yarn roll’s center detected by Yolo is not accurate; therefore, we detect the bobbin’s center by circle filters. Circle filters are designed as shown in Figure 7a,b—their diameter can be changed. Here, we design some circle_x and circle_y according to the bobbin’s pixel diameter. Then, we process the restored ROI image via the following steps.

**Figure 8:**
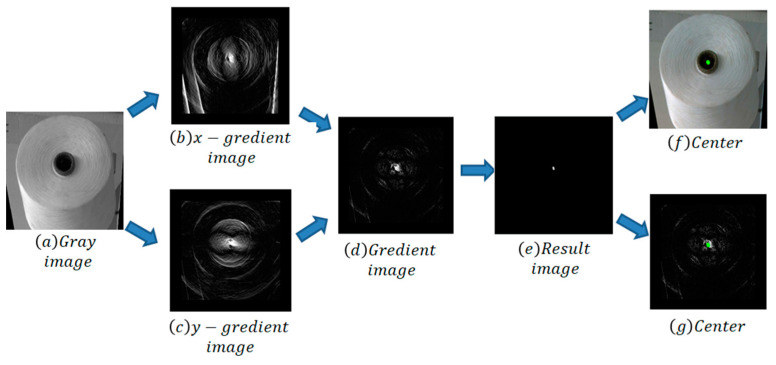
Center detection process.

(1) Transform raw image into gray image;
(2) Design circle-filters with different diameter according to the circle’s features in the image. Figure 7a,b show two circle filters designed to detect a circle of seven pixels in diameter. The bobbin’s diameter is about 60–100 pixels, so we should design four circle_x and circle_y with diameter 50 and 30 pixels;
(3) Obtain convolution image by sliding circle_x in the gray image with a fixed step to the computer convolution and geting x-gradient image, then getting y-gradient image in the same way. X-gradient and y-gradient images are shown in Figure 8b,c;
(4) Use AND operation to process corresponding pixel values of the x-gradient image and the y-gradient image and obtain the gradient image, which is shown in Figure 8d;
(5) Use “erode” and “dilate” methods to process the gradient image and obtain the result image, which is shown in Figure 8e;
(6) Detect contours in the result image. The max contour is the yarn roll’s center, drawn in the raw and gradient images, which are shown in Figure 8f,g.

#### 3.2.3. Extracting the Yarn Roll’s Contours

Following the above steps, we can restore every ROI image and obtain the bobbin’s center in the image. Next, we detect the yarn roll’s diameter according to the circle’s features. We process the image by following the steps below.

**Figure 9:**
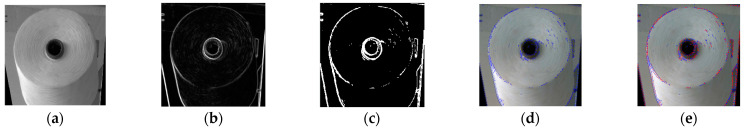
Image process and contour points extraction. (**a**) Gray image. (**b**) Gradient image. (**c**) Binary image. (**d**) Contour points. (**e**) Points interpolation.

(1) We transform the RGB image into a gray image, which is shown in Figure 9a;
(2) We process the gray image with the sobel filter [13] and obtain the gradient image, which is shown in Figure 9b. The sobel filter can eliminate the influence of light and color;
(3) We convert the gradient image into a binary image, which is shown in Figure 9c;
(4) We use opencv’s “findContours” founction to process binary image to get contour points, which is shown in Figure 9d, blue points are extracted from binary image;
(5) We can see from Figure 9d that contour points are dense in a zigzag fashion, but sparse in the smooth place. In order to obtain a good detection result, we need to insert points in the sparse place, which is shown in Figure 9e. Red points are the inserted points.

**Figure 10:**
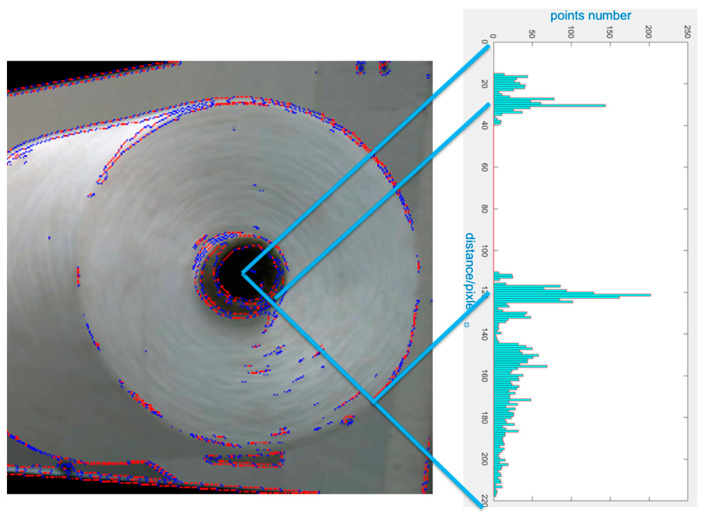
Histogram of distance distribution between contour points and bobbin’s center.

We can see from Figure 9 that the yarn roll’s contour is intermittent and the contour points are disorderly; therefore, it is difficult to extract the yarn roll’s end surface directly. However, following the above steps, we obtain the bobbin’s center in the image and the yarn roll’s end surface corresponds to the circle. Therefore, we use the circle’s feature (all points on a circle are equidistant from the center) to detect the yarn roll. We calculate the distance between every contour point and the bobbin’s center, then we draw a histogram, which is shown in Figure 10. The horizontal axis is the distance between contour points and the central point of the bobbin in the pixels; the vertical axis is the number of points with the same distance. It can be seen from the histogram that there are two peaks, the first peak corresponds to the pixel radius of the bobbin, and the second peak corresponds to the pixel radius of the yarn roll’s outer edge.

**Figure 11:**
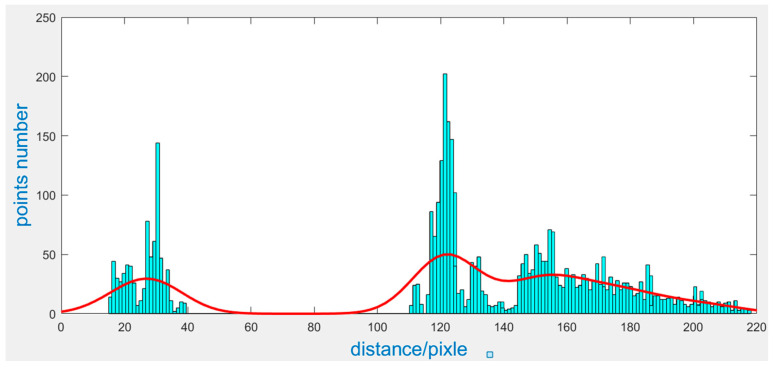
Kernel density estimation curve.

The drawing and calculation of the histogram have been completed. In the application, it is necessary to calculate the peak position of the histogram. Therefore, the histogram is fitted, and the peak points are calculated by the fitting function. Here, the kernel density estimation method is used to obtain a smooth density curve. The formula of the kernel density estimation is shown in Formula (4), where K is the kernel function [38], and a Gaussian function [39] is generally selected as the kernel function, which is shown in Formula (5), N is the total number of samples, and h is the bandwidth. In general, the value of h is 1. This way, the distance density curve can be obtained, as shown in Figure 11. Then, the maximum value of the curve can be calculated by derivation. If the maximum of the curve corresponds to the peak value of the histogram (distance difference is no more than five pixels), the distance corresponding to the peak value can be considered as the pixel radius of the bobbin and yarn roll.

(4) $$f\left(x,h\right)=\frac{1}{Nh}\overset{N}{\underset{i=1}{\sum }}K\left(\frac{x-x_{i}}{h}\right)$$

(5) $$K\left(u\right)=\frac{1}{\sqrt{2\pi }}e^{-\frac{u^{2}}{2}}$$

**Figure 12:**
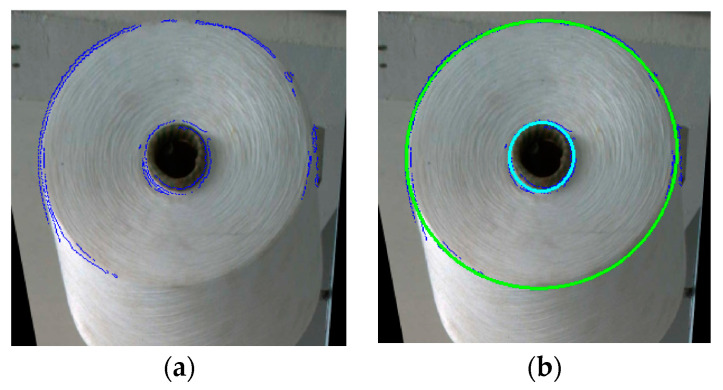
Points sifted and ellipse fitting. (**a**) Points after sifted. (**b**) Ellipse fitting.

The above steps have yielded a more precise yarn roll and bobbin radius (pixel value). However, it is necessary to sift and fit points to improve the detection accuracy. After the approximate radius is obtained, the points within a certain distance range (plus or minus five pixels) are highlighted, as shown in Figure 12a. Then, the remaining points are fitted using the ellipse fitting formula, which is shown in Figure 12b. The diameter pixels of the bobbin and yarn roll are obtained.

### 3.3. Fusing Diameter Detected by Yolo and Contours

Following the above steps, the yarn roll’s and bobbin’s diameters are obtained from the Yolo and contours, respectively. Both methods have their advantages and disadvantages. There is barely any false detection in Yolo. However, the boxes detected by Yolo are often larger and smaller compared with the yarn roll’s and bobbin’s diameter, respectively. Compared with Yolo, the contours method is more accurate, but this method is not suitable when the yarn roll’s margin is low. Therefore, we need to fuse the corresponding results. The method is as follows.

(1) A weight value can be calculated. First, the maximum value of the density curve must be obtained through the above steps. Then, the peak of the corresponding histogram is found around the maximum value of the curve (plus or minus five pixels). Second, the average value of the histogram is calculated. Third, the weight value μ is calculated using Equation (6), *m* is the mean value of the histogram and p is the peak value of the histogram.
  (6) $$\mu =\left\{\begin{array}{c} 4m/p\;\;\;\;\;\;if\;4m/p<1\;\\ 1\;\;\;\;\;\;\;\;\;\;if\;4m/p\geq 1 \end{array}\right.$$
(2) The weighted average is calculated using Equation (7), *r* is the bobbin’s radius, $r_{1}$ is the bobbin’s radius detected by Yolo, $r_{1}$ is the bobbin’s radius detected by contours, *R* is the yarn roll’s radius.
  (7) $$\left\{\begin{array}{c} r=\left(1-\mu \right)r_{1}+\mu r_{2}\\ R=\left(1-\mu \right)R_{1}+\mu R_{2} \end{array}\right.$$

### 3.4. Calculating the Yarn Roll’s Diameter and Length in Real World

After the diameter in pixel is found for both the bobbin and the yarn roll, the bobbin’s diameter in the real world can be obtained (the cylindrical bobbin and conic bobbin can be easily discriminated in the image). We use Formula (8) to calculate the yarn roll’s diameter in the real world, d is the bobbin’s diameter in the real world, $d^{\prime }$ is the bobbin’s diameter in the image, D is the yarn roll’s diameter in the real world, $D^{\prime }$ is the yarn roll’s diameter in the image.

(8) $$\frac{d^{\prime }}{d}=\frac{D^{\prime }}{D}$$

**Figure 13:**
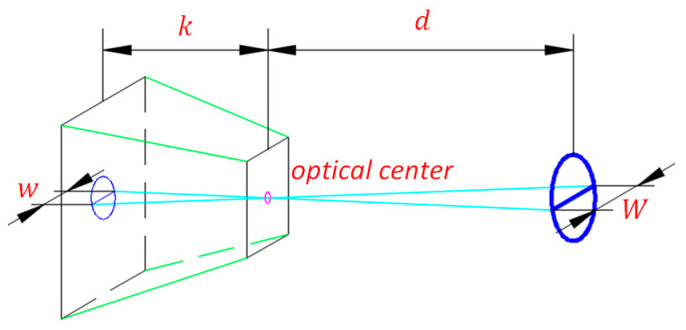
Camera model.

After that, we calculate the distance between the yarn roll’s end face and the camera according to the distance detection theory based on monocular vision technique, which is shown in Figure 13. The distance from the camera and the yarn roll’s end face is d, the camera’s focal length is k, the yarn roll’s diameter in the real world is W, the yarn roll’s diameter in the image is w. Therefore, we can obtain d by applying the formula: k/d=w/W. Because the distance between the yarn roll’s support and the camera is known, the difference value is the yarn roll’s length. Because the conic yarn roll’s length is known, we only use this method to discriminate the detected cylindrical yarn roll.

### 3.5. Kalman Filter

After calculating the main size of the yarn roll, we can use Formula (1) or Formula (2) to calculate the yarn roll’s margin. We use the Kalman filter [40] to prevent false detection from occurring at the same time: (1) we predict the yarn roll’s theoretical margin according to the yarn consumption speed and time; (2) we fuse the predicted value and the measured value (obtained using the abovementioned computer vision method) using the Kalman filter. The main formula is shown from Formula (9) to Formula (14). In Formula (9), it is assumed that the time interval is t and that the yarn roll’s margin and yarn consumption speed at the last detection time is $value_{k-1}$ and $v_{k-1}$, respectively. $value_{k}$ and $v_{k}$ are the current margin and the current speed. u is the variation of yarn consumption speed. In Formula (11), P is the covariance matrix of yarn consumption speed and margin. Q is the noise matrix. In Formula (12), H = [1,0] is the observation matrix. In Formula (13), $Z_{k}$ is the measured value of the yarn roll’s margin.

(9) $$x_{k}^{-}=\left[\begin{array}{c} value_{k}\\ v_{k} \end{array}\right]=F\left[\begin{array}{c} value_{k-1}\\ v_{k-1} \end{array}\right]+Bu$$

(10) $$F=\left[\begin{array}{cc} 1 & -t\\ 0 & 1 \end{array}\right]\;\;\;\;\;\;\;\;\;\;B=\left[\begin{array}{c} -0.5t^{2}\\ t \end{array}\right]$$

(11) $$P_{k}^{-}=FP_{k-1}F^{T}+Q$$

(12) $$K_{k}=P_{k}^{-}H^{T}{\left(HP_{k}^{-}H^{T}+R\right)}^{-1}$$

(13) $$x_{k}=x_{k}^{-}+K_{k}\left(Z_{k}-Hx_{k}^{-}\right)$$

(14) $$P_{k}=\left(I-K_{k}H\right)P_{k}^{-}$$

## 4. Experiments

Some experiments have been designed to test the method. The first experiment entails testing the accuracy of the yarn roll’s diameter estimate. The second experiment entails testing the accuracy of the yarn roll’s length estimate. The third experiment entails testing the effect of the Kalman filter. The fourth experiment entails testing the accuracy of the yarn roll detection.

**Figure 14:**
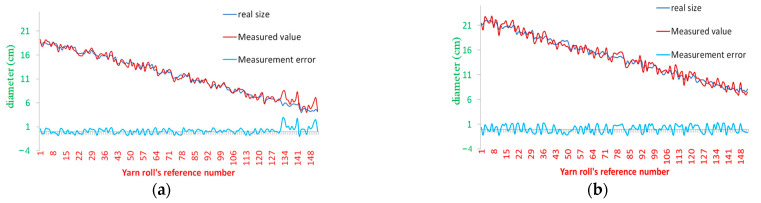
Yarn roll’s diameter measurement error. (**a**) Conic yarn roll. (**b**) Cylindrical yarn roll.

In the first experiment, we take 150 conic yarn rolls with different diameter. Then, we sort them according to the yarn roll’s diameter and provide a reference number. Thirdly, we measure every yarn roll’s diameter using vernier caliper as ground truth, which is illustrated by the dark blue line in Figure 14a. Fourthly, we use our method to calculate the yarn roll’s diameter from the image, which is illustrated by the red line in Figure 14a. Lastly, we calculate the measurement error, which is illustrated by the light blue line in Figure 14a. The cylindrical yarn roll’s data are presented in Figure 14b. From Figure 14 we can see that the measurement error of the conical yarn roll’s diameter is no more than 10% (6 mm) when its diameter is larger than 6 cm. When its diameter is less than 6 cm, the measurement error is very large. The reason for that is that Yolo and the contours method are poor. The measurement error of the cylindrical yarn roll’s diameter is no more than 15.4% (12 mm). The measurement error of the yarn roll’s diameter is 3.9% (8.6 mm).

**Figure 15:**
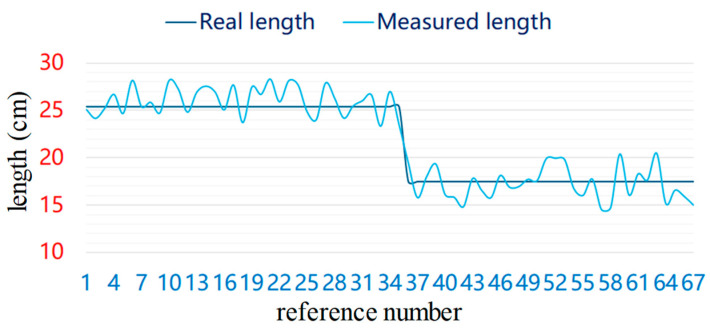
Yarn roll’s length measurement error.

In the second experiment, 67 long and short cylindrical rolls of yarn were placed on a holder and numbered in order. The length of each yarn roll is measured using the method described above and is illustrated as a light blue curve in Figure 14a. The calculation of the diameter of the yarn roll is performed using the above method and is represented by a red line in Figure 15. The measurement error is no more than 4 cm, the difference between long cylindrical yarn roll’s length and long cylindrical yarn roll’s length is 10.5 cm. Therefore, this method can discriminate the detected cylindrical yarn roll.

**Figure 16:**
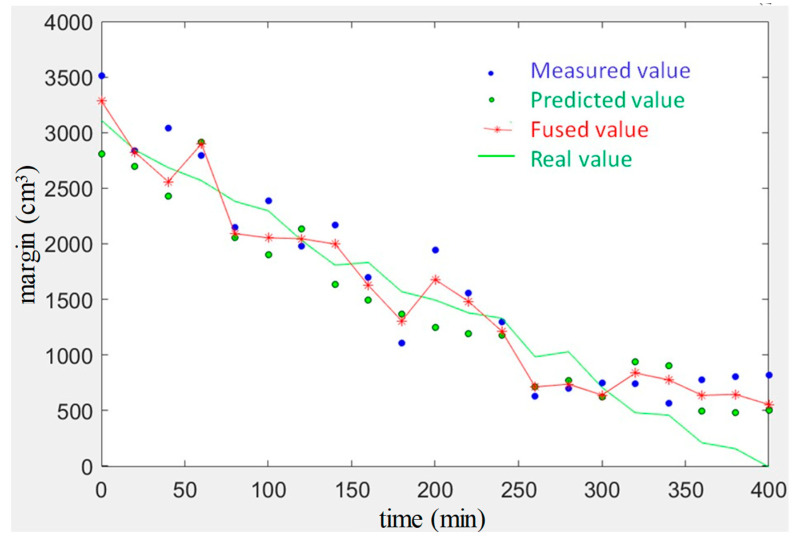
Kalman filter effect.

In the third experiment, the effect of the Kalman filter is verified by detecting a yarn roll’s margin every 20 min. The final result is shown in Figure 16. The blue point is the measured value and is calculated according to the image, the green point is the predicted value of the yarn roll’s margin, the green line is the actual value of the yarn roll’s margin, which is measured and calculated by vernier caliper, and the red line is the final result fused using the Kalman filter. It can be seen that the Kalman filter can improve accuracy to a certain extent, especially when the yarn roll’s margin is small, reducing detection error.

**Figure 17:**
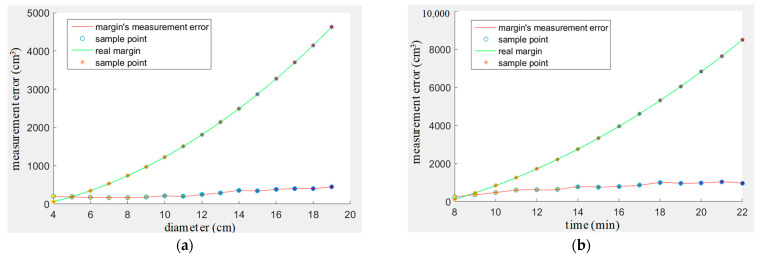
Yarn roll’s margin measurement error. (**a**) Conic yarn roll. (**b**) Cylindrical yarn roll.

In the fourth experiment, Formulas (1) and (2) are used to calculate the yarn roll’s margin. During the calculation, the measurement error of the diameter will be magnified. Here, the error value of the margin is not used as an evaluation index, but only for statistics. For the cylindrical yarn roll, ten cylindrical yarns with diameters of 22 cm, 21 cm, 20 cm, etc., are taken and the margin calculated as a measured value through the above method. Then, we predict the current margin according to the yarn consumption speed and combine the two values through the Kalman filter as the final result. Figure 17a shows the detection error of long cylindrical yarn roll margin, and there are ten groups of data for which the average error is calculated at each sampling point. The conical yarn roll is also sampled and measured. The final error is shown in Figure 17b. The errors are all absolute values, so there are no negative numbers.

## 5. Discussion

This method can detect the yarn roll’s margin online through monocular camera, which can initially replace manual inspection and detection. This system initially meets the requirements of enterprises. It is innovative in its algorithms, it has good adaptability, and it is easy to be deployed at different sites. However, through early application, it is possible to observe that this method can be further improved. Suggestions are listed as follows.

(1) The method is based on monocular camera. The measurement error of the yarn roll’s length is large, its error being about 3 cm. Since the types of yarn roll in this enterprise are few, the measurement accuracy will not be affected temporarily. If more types of yarn roll with different diameter and lengths are used, a stereo camera needs to be considered. The latter’s accuracy is higher, and so is the price;
(2) The detection algorithm should be optimized, especially to improve the detection accuracy when the yarn roll’s margin is small. The existing algorithm presents some defects;
(3) The resolution of the camera can be increased. Increasing resolution can improve detection accuracy, but the detection speed will decrease correspondingly.

## Data Availability

Not applicable.
